# Multidimensional comparative evaluation of first-line therapies for extensive-stage small cell lung cancer: a systematic review and network meta-analysis of clinical efficacy and safety profiles

**DOI:** 10.1186/s12885-025-14750-4

**Published:** 2025-08-09

**Authors:** Ziyao Jiang, Fangrui Zhao, Butuo Li, Junyi He, Huiwen Yang, Yuhan Ji, Bing Zou, Jinming Yu, Linlin Wang

**Affiliations:** 1 Department of Radiation Oncology, Shandong Cancer Hospital and Institute, Shandong First Medical University, Shandong Academy of Medical Sciences, Jinan, 250117 Shandong China; 2 Department of Oncology, Shandong Cancer Hospital and Institute, Shandong First Medical University, Shandong Academy of Medical Sciences, Jinan, 250117 Shandong China; 3 Cheeloo College of Medicine, Shandong University Cancer Center, Shandong University, Jinan, 250117 Shandong China

**Keywords:** Small cell lung cancer, Extensive-stage, Chemoimmunotherapy, Immune checkpoint inhibitor, Angiogenesis, Network meta-analysis

## Abstract

**Background:**

The first-line treatment for extensive-stage small cell lung cancer (ES-SCLC) has evolved from chemotherapy alone to chemoimmunotherapy. However, the improvements in overall survival (OS) and progression-free survival (PFS) have been modest. Therefore, this study employs a comprehensive multidimensional evaluation framework to identify optimized therapeutic combinations with enhanced efficacy and improved safety profiles in the immunotherapy era.

**Methods:**

An adaptive search strategy was employed to retrieve all relevant literature from electronic databases, including PubMed, Embase, Web of Science, and the Cochrane Library, from database inception to November 2024. The retrieved studies were carefully screened according to pre-designed inclusion and exclusion criteria. Clinical research articles and their supplementary materials that met the criteria were obtained and thoroughly reviewed. Manual data extraction was conducted, with the safety data and efficacy outcomes. A network meta-analysis of all acquired data for each outcome was performed. The study protocol was pre-registered in PROSPERO, CRD42024612944.

**Results:**

This network meta-analysis included 6,473 patients from 14 head-to-head randomized controlled trials (RCTs). Compared with etoposide-platinum chemotherapy combined with a programmed cell death ligand 1 inhibitor (the Chemo + PD-L1 regimen), the addition of anlotinib (the Chemo + PD-L1 + Anlo regimen) resulted in better PFS (hazard ratio (HR), 0.42; 95% confidence interval (CI), 0.33–0.54) and objective response rate (ORR) (odds ratio (OR), 1.81; 95% CI, 1.13–2.91). Moreover, adding BMS-986012 (anti-fucosyl-GM1 antibodies) to the Chemo + PD-L1 regimen ranked first in the surface under the cumulative ranking curve (SUCRA, 0.96) analysis for OS. Compared with the Chemo + PD-L1 regimen, the addition of an anti-CTLA-4 inhibitor (the Chemo + PD-L1 + CTLA-4 regimen) was associated with an increased risk of treatment-related adverse events (TRAEs) of grades ≥ 3 (risk ratio (RR), 1.19; 95%CI, 1.04–1.36).

**Conclusions:**

Incorporating anlotinib into the Chemo + PD-L1 regimen can be a viable first-line option for patients with high tumor burden, but cannot fully replace the current first-line standard-of-care (SOC). Chemoimmunotherapy combined with immune-related targeting drugs demonstrates the potential to improve overall survival.

**Supplementary Information:**

The online version contains supplementary material available at 10.1186/s12885-025-14750-4.

## Introduction

Small cell lung cancer (SCLC) is a highly aggressive malignancy, representing approximately 15% of all lung cancer cases [1]. Patients with SCLC often experience rapid tumor metastasis in the early stages, leading to an estimated 70% of patients presenting with ES-SCLC at the time of initial diagnosis [2, 3]. Over the past few decades, the first-line SOC for ES-SCLC has primarily been etoposide-platinum chemotherapy [4] (the Chemo regimen), with a median OS or PFS generally not exceeding 1 year [1, 4] or 5 months, respectively. The addition of immunotherapy has somewhat broken this stagnation. Since the introduction of PD-1 and PD-L1 inhibitors, the current first-line chemoimmunotherapy has finally improved survival data in terms of OS and PFS for ES-SCLC patients [5]. However, this improvement is modest, extending OS or PFS by only 2–3 months or less than half a month [6], respectively. Exploring and innovating first-line treatment modalities for patients with ES-SCLC is essential.

Currently, medium- to large-scale randomized controlled trials (RCTs) predominantly focus on investigating novel add-on therapies to first-line chemoimmunotherapy [7–10]. These emerging strategies include combining the current first-line SOC with an anti-angiogenic agent [8, 10] (macromolecular or small molecular), another immune checkpoint inhibitor [7, 11] (anti-CTLA-4 or anti-TIGIT antibodies), or an immune-related targeting drug [9] (anti-fucosyl-GM1 antibodies).

Since chemoimmunotherapy became the SOC, there are no network meta-analyses that have systematically included and evaluated these chemoimmunotherapy-based regimens. Existing pairwise meta-analyses [12–14] remain mostly confined to comparisons between chemoimmunotherapy and chemotherapy alone, with outdated evidence needing updating following recent RCT publications [10, 15–17]. Furthermore, conventional pairwise meta-analysis frameworks are inadequate for addressing the multidirectional exploration of first-line therapeutic strategies, which necessitates multiple comparisons across diverse treatment modalities.

Thus, we systematically summarized the traditional chemotherapy regimen, the current first-line SOC chemoimmunotherapy, and its novel derivative therapeutic strategies to inform clinical guideline updates and future research directions. These regimens were subjected to Bayesian network meta-analysis and ranked after surface under the cumulative ranking curve (SUCRA) analysis. A multidimensional comparative evaluation was performed to assess these therapeutic approaches in terms of clinical efficacy and safety profiles.

## Methods

The network meta-analysis was conducted per the Preferred Reporting Items for Systematic Reviews and Meta-Analyses (PRISMA) guidelines and the PRISMA extension statement. Inclusion and exclusion criteria were first established, followed by the execution of five key processes: literature search, study selection, assessment of quality, data extraction, and statistical analysis of data. To ensure transparency, foresight, and innovation in the research process, the study protocol was pre-registered in the International Prospective Register of Systematic Reviews (PROSPERO) under registration number CRD42024612944.

### Data sources and search strategy

From the database inception to November 2024, we conducted a comprehensive search of published relevant literature in the PubMed, Embase, Web of Science, and Cochrane Library databases. Additionally, the reference lists of the included studies and relevant reviews were manually searched to finalize the selection. The search strategy consisted of three main components: disease name (small cell lung cancer), disease stage (extensive stage), and clinical trial type (randomized controlled trial). Each component used a combination of subject headings and keywords, with the keywords tailored according to the thematic categories of each database. Appropriate Boolean operators were employed to establish logical connections between subject headings and keywords within individual components, as well as across distinct components. The detailed search strategy is provided in Supplementary File 1.

### Study selection

Studies were eligible for inclusion if they (1) were Phase II/III head-to-head RCTs; (2) enrolled participants with histologically or cytologically confirmed ES-SCLC (according to the Veterans Administration Lung Study Group staging system), who had not previously received systemic anticancer therapy; (3) had a comparative design with ≥ 2 treatment arms, including at least one chemoimmunotherapy regimen (chemotherapy + immune checkpoint inhibitor); and (4) provided complete data for OS and PFS, particularly the hazard ratios (HRs) with 95% CI for comparisons, as well as aimed to meet the requirement of providing data for ORR, disease control rate (DCR), and safety data whenever possible. Trials not fulfilling all four criteria were excluded.

OS refers to the time from randomization to death due to any cause. PFS is the time from randomization to disease progression or death due to any cause, whichever occurs first. ORR refers to the sum of the proportions of patients with complete response (CR) and partial response (PR). DCR refers to the sum of the proportions of patients with CR, PR, and stable disease (SD). Safety data focus on the incidence of treatment-related adverse events (TRAEs), especially specific types of adverse events (AEs), including leukopenia, neutropenia, anemia, and vomiting.

### Quality assessment

Two researchers (ZJ and FZ) independently conducted a risk of bias assessment for all included studies using the criteria of the Cochrane Collaboration Risk of Bias Tool. The evaluation focused on five main domains: the randomization process, deviations from intended interventions, missing outcome data, measurement of the outcome, and selection of the reported result. After considering the performance of each study in these five areas, an overall assessment was provided. The final decision was based on the consensus between the two reviewers, with each study receiving a risk of bias rating of “high,” “unclear,” or “low”.

### Data extraction

Following the PRISMA guidelines, detailed data extraction was conducted for all included studies. To ensure accuracy and comprehensiveness, data on clinical trial identification, baseline population characteristics, and clinical outcomes were extracted and summarized collaboratively by three authors (HY, YJ, and BZ). Any discrepancies were resolved through a thorough discussion with a fourth author (LW) to reach a consensus.

The clinical trial identification information included the study name, the clinical trial registration number, authors, publication year, study phase, treatment regimen, and the number of participants. Baseline population characteristics included patient age, gender distribution, performance status, and metastasis to major organs (data on the association between population characteristics and outcome measures could not be collected). For clinical outcomes, the primary survival data included PFS and OS, with HR and 95%CI extracted. Corresponding data for ORR and DCR were also recorded. Data on the aggregate incidence of grade ≥ 3 TRAEs and specific incidence rates of common TRAEs—including diarrhea, nausea, vomiting, leukopenia, neutropenia, thrombocytopenia, and anemia—were extracted from studies. When these data were unavailable, AE data were included instead.

Information from the original and updated studies, supplementary materials, conference proceedings, and ClinicalTrials.gov were aggregated to obtain the most comprehensive and up-to-date data.

### Statistical analysis of data

Using JAGS software in conjunction with the gemtc package in R software (version 4.2.1), a network meta-analysis was conducted within a Bayesian framework using the MCMC method. Four Markov chains were used for the simulation, with an initial value of 2.5 and a thinning interval of 1. The pre-simulation burn-in phase consisted of 20,000 iterations for annealing, followed by 50,000 iterations to ensure model convergence. The deviance information criterion (DIC) was used to assess the relative goodness-of-fit between fixed-effect and random-effect models, with a lower DIC value indicating better model fit. If the DIC difference was ˂ 5, consistency between the fixed-effect and random-effect models was confirmed.

The HR was used to measure the effect size for survival outcomes (OS and PFS), the OR for binary outcomes (ORR and DCR), and the risk ratio (RR) for safety outcomes (TRAEs), all with their corresponding 95%CI. Effect sizes were considered statistically significant if the 95%CI did not include 1 or if the two-sided *P* value was < 0.05. In specific comparative analyses, experimental therapeutic combinations with an HR < 1 for OS or PFS, or an OR > 1 for ORR or DCR, were considered clinically superior to control regimens. An RR > 1 for TRAEs indicated a higher toxicity risk in the experimental combination.

The analysis results included a network plot and the league table for each outcome measure. Additionally, for more intuitive results, the SUCRA value was used as an indicator of cumulative ranking probability. Therapeutic intervention with a SUCRA score of 1 definitively holds the highest hierarchical position among all treatment groups, whereas a value approaching 0 indicates the lowest ranking. Heatmaps were used to visually compare SUCRA values across treatment groups.

Statistical analysis was conducted by two researchers (HY and YJ), and the results were cross-validated by three additional researchers (FZ, BL, and JH) to ensure accuracy.

## Results

### Systematic review and the characteristics of all trials

**Fig. 1:**
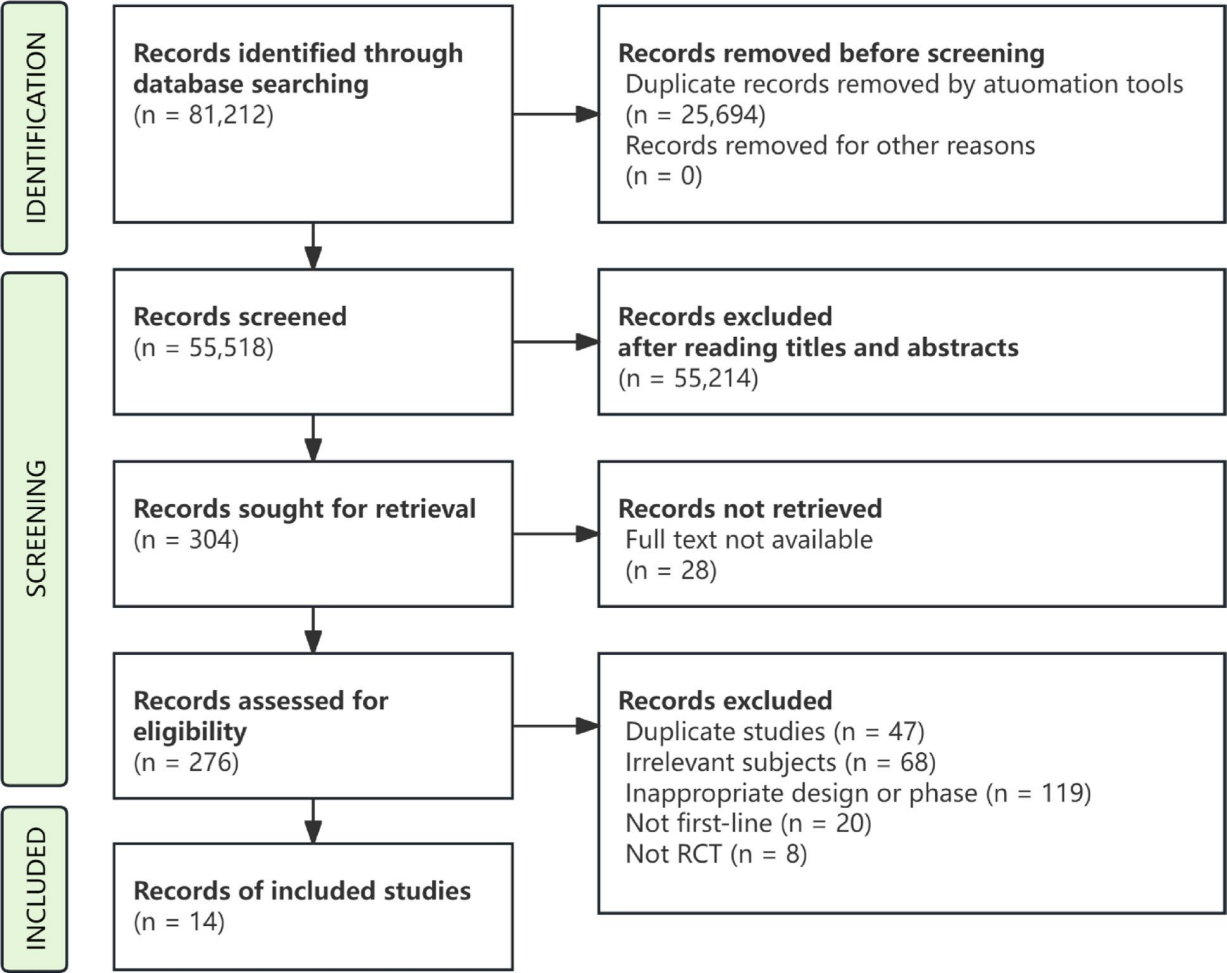
The Flowchart of Literature Screening Process

Following comprehensive database interrogation, 81,212 records were initially identified for preliminary screening. After duplicate removal (*n* = 25,694), 55,518 citations underwent title/abstract evaluation, resulting in 304 potentially relevant articles proceeding to the full-text review. Through independent full-text assessment and further detailed evaluation, 14 studies were selected based on the inclusion and exclusion criteria. The process of study selection is illustrated in Fig. 1. The included studies comprised 3 Phase II RCTs and 11 Phase III RCTs. The baseline characteristics of each study are summarized in Table 1.

**Table 1:** Baseline characteristics of all trials included in the network Meta-Analysis for patients with ES-SCLC

Source(Name)	Trial registration number	Phase	Design	Treatment	Participants, No.	Gender, Male No./Female No.	Age, <65 No./total No.(%)	ECOG PS, 0 No./1 No.	Brain or CNS metastases No./total No.(%)	Liver metastases No./total No.(%)	Reported Outcomes
Cheng [10] (ETER701)	NCT04234607	3	RCT, double-blind, placebo-controlled, multicenter	Etoposide plus Carboplatin plus Benmelstobart plus Anlotinib	246	209/37	150/246 (60.98)	47/199	25/246 (10.16)	79/246 (32.11)	OS, PFS, ORR, DCR, Safety outcomes
				Etoposide plus Carboplatin	247	207/40	147/247 (59.51)	48/199	26/247 (10.53)	79/247 (31.98)	
Cheng et al. [16] (RATIONALE-312)	NCT04005716	3	RCT, double-blind, placebo-controlled, multicenter	Etoposide-platinum chemotherapy ^**a**^ plus Tislelizumab	227	186/41	138/227 (60.79)	35/192	1/227 (0.44)	64/227 (28.19)	OS, PFS, ORR, DCR, Safety outcomes
				Etoposide-platinum chemotherapy	230	186/44	149/230 (64.78)	34/196	4/230 (1.74)	59/230 (25.65)	
Rudin et al. [7] (SKYSCRAPER-02)	NCT04256421	3	RCT, double-blind, placebo-controlled, multicenter	Etoposide plus Carboplatin plus Atezolizumab plus Tiragolumab	243	162/81	117/243 (48.15)	86/156	47/243 (19.34)	89/243 (36.63)	OS, PFS, ORR, DCR, Safety outcomes
				Etoposide plus Carboplatin plus Atezolizumab	247	164/83	116/247 (46.96)	82/165	46/247 (18.62)	94/247 (38.06)	
Ewa et al. (2024) (No name)	NCT04702880	2	RCT, open-label multicenter	Etoposide plus Carboplatin plus Nivolumab plus BMS-986012	67	43/24	26/67 (38.81)	16/51	21/67 (31.34)	29/67 (43.28)	OS, PFS, ORR, DCR, Safety outcomes
				Etoposide plus Carboplatin plus Nivolumab	66	33/33	26/66 (39.39)	17/49	17/66 (25.76)	28/66 (42.42)	
Ohe et al. [8] (BEAT-SC)	jRCT2080224946	3	RCT, open-label multicenter	Etoposide-platinum chemotherapy plus Atezolizumab plus Bevacizumab	167	138/29	NR	48/119	38/167 (22.75)	NR	OS, PFS, ORR, DCR, Safety outcomes
				Etoposide-platinum chemotherapy plus Atezolizumab	166	137/29	NR	47/119	27/166 (16.27)	NR	
Cheng et al. [17] (EXTENTORCH)	NCT04012606	3	RCT, double-blind, placebo-controlled, multicenter	Etoposide-platinum chemotherapy plus Toripalimab	223	183/40	144/223 (64.57)	42/181	3/223 (1.35)	60/223 (26.91)	OS, PFS, ORR, DCR, Safety outcomes
				Etoposide-platinum chemotherapy	219	183/86	124/219 (56.62)	38/181	4/219 (1.83)	50/219 (22.83)	
Cheng et al. (2022) (ASTRUM-005)	NCT04063163	3	RCT, double-blind, placebo-controlled, multicenter	Etoposide plus Carboplatin plus Serplulimab	389	317/72	235/389 (60.41)	71/318	50/389 (12.85)	99/389 (25.45)	OS, PFS, ORR, DCR, Safety outcomes
				Etoposide plus Carboplatin	196	164/32	119/196 (60.71)	32/164	28/196 (14.29)	51/196 (26.02)	
Wang et al. [3] (CAPSTONE-1)	NCT03711305	3	RCT, double-blind, placebo-controlled, multicenter	Etoposide plus Carboplatin plus Adebrelimab	230	184/46	155/230 (67.39)	33/197	5/230 (2.17)	73/230 (31.74)	OS, PFS, ORR, DCR, Safety outcomes
				Etoposide plus Carboplatin	232	188/44	147/232 (63.36)	30/202	5/232 (2.16)	74/232 (31.90)	
Besse et al. (2020) (REACTION)	NCT02580994	2	RCT, open-label multicenter	Etoposide-platinum chemotherapy plus Pembrolizumab	61	44/17	NR	20/38	5/61 (8.20)	NR	OS, PFS, ORR, Safety outcomes
				Etoposide-platinum chemotherapy	64	36/28	NR	24/37	7/64 (10.94)	NR	
Leal et al. (2020) (ECOG-ACRIN EA5161)	NCT03382561	2	RCT, open-label multicenter	Etoposide-platinum chemotherapy plus Nivolumab	80	35/45	NR	23/57	NR	NR	OS, PFS, ORR, Safety outcomes
				Etoposide-platinum chemotherapy	80	36/44	NR	24/56	NR	NR	
Rudin et al. (2020) (KEYNOTE-604)	NCT03066778	3	RCT, double-blind, placebo-controlled, multicenter	Etoposide-platinum chemotherapy plus Pembrolizumab	228	152/76	115/228 (50.44)	60/168	33/228 (14.47)	95/228 (41.67)	OS, PFS, ORR, DCR, Safety outcomes
				Etoposide-platinum chemotherapy	225	142/83	101/225 (44.89)	56/169	22/225 (9.78)	92/225 (40.89)	
Paz-Ares et al. [11] (CASPIAN)	NCT03043872	3	RCT, open-label multicenter	Etoposide-platinum chemotherapy plus Durvalumab plus Tremelimumab	268	202/66	154/268 (57.46)	109/159 ^**b**^	38/268 (14.18)	117/268 (43.66)	OS, PFS, ORR, DCR, Safety outcomes
				Etoposide-platinum chemotherapy plus Durvalumab	268	190/78	167/268 (62.31)	99/169 ^**b**^	28/268 (10.45)	108/268 (40.30)	
				Etoposide-platinum chemotherapy	269	184/85	157/269 (58.36)	90/179 ^**b**^	27/269 (10.04)	104/269 (38.66)	
Horn et al. (2018) (IMPOWER133)	NCT02763579	3	RCT, double-blind, placebo-controlled, multicenter	Etoposide plus Carboplatin plus Atezolizumab	201	129/72	111/201 (55.22)	73/128	17/201 (8.46)	NR	OS, PFS, ORR, Safety outcomes
				Etoposide plus Carboplatin	202	132/70	106/202 (52.48)	67/135	18/202 (8.91)	NR	
Reck et al. [24] (No name)	NCT01450761	3	RCT, open-label multicenter	Etoposide-platinum chemotherapy plus Ipilimumab	566	317/161	299/566 (52.83)	137/340	55/566 (9.72)	NR	OS, PFS, ORR, DCR, Safety outcomes
				Etoposide-platinum chemotherapy	566	326/150	277/566 (48.94)	147/328	45/566 (7.95)	NR	

#1. a The choice of platinum, either cisplatin or carboplatin, will be designated by the doctor according to the specific condition of the patient

b WHO performance status (ECOG PS was not used to assess patients’ performance status in the study.)

#2. Abbreviations: NR, Not Reported; ECOG PS, Eastern Cooperative Oncology Group Performance Status; CNS, Central Nervous System; RCT, Randomized Controlled Trial; ES-SCLC, Extensive-Stage Small Cell Lung Cancer; OS, Overall Survival; PFS Progression-free Survival; ORR, Objective Response Rate; DCR, Disease Control Rate

### Classification of treatment modalities and the composition of the network

Overall, a total of 6,473 patients were enrolled in this analysis, and treatment modalities were organized and classified into the following categories (Supplementary Fig S1 Graphical Abstract).

- One conventional first-line SOC—etoposide-platinum chemotherapy (the Chemo regimen).
- Three immune checkpoint inhibitors (ICIs) combined with the Chemo regimen:
  - The Chemo + PD-L1 regimen.
  - The Chemo + PD-1 regimen.
  - Etoposide-platinum chemotherapy combined with an anti-CTLA-4 inhibitor (the Chemo + CTLA-4 regimen).
- The Chemo + PD-1 regimen, with the addition of a novel immune-related targeting therapy—anti-fucosyl-GM1 antibody (BMS-986012) (the Chemo + PD-1 + BMS regimen).
- The Chemo + PD-L1 regimen, with the addition of four different drugs:
  - Anlotinib (the Chemo + PD-L1 + Anlo regimen).
  - Bevacizumab (the Chemo + PD-L1 + Bev regimen).
  - Anti-CTLA-4 inhibitor (the Chemo + PD-L1 + CTLA-4 regimen) or.
  - Anti-TIGIT inhibitor (the Chemo + PD-L1 + TIGIT regimen).

Then a network was built to allow for multiple comparisons of these regimens (Fig S2).

### Risk of bias in the included studies

The evaluation results from both researchers are shown in Supplementary Figure S3. In the areas of Randomization Process and Deviations from Intended Interventions, one article was considered to have a “high risk” each, with three articles in each area rated as having “some concerns.” In the domains of Missing Outcome Data and Selection of the Reported Result, two studies were each identified as having “some concerns.” Additionally, in terms of Measurement of the Outcome, three studies were deemed to have “some concerns.” Overall, two articles were classified as “high risk,” two as having “some concerns,” and the remaining articles were classified as “low risk.”

### Network meta-analysis for the outcomes of efficacy

**Fig. 2:**
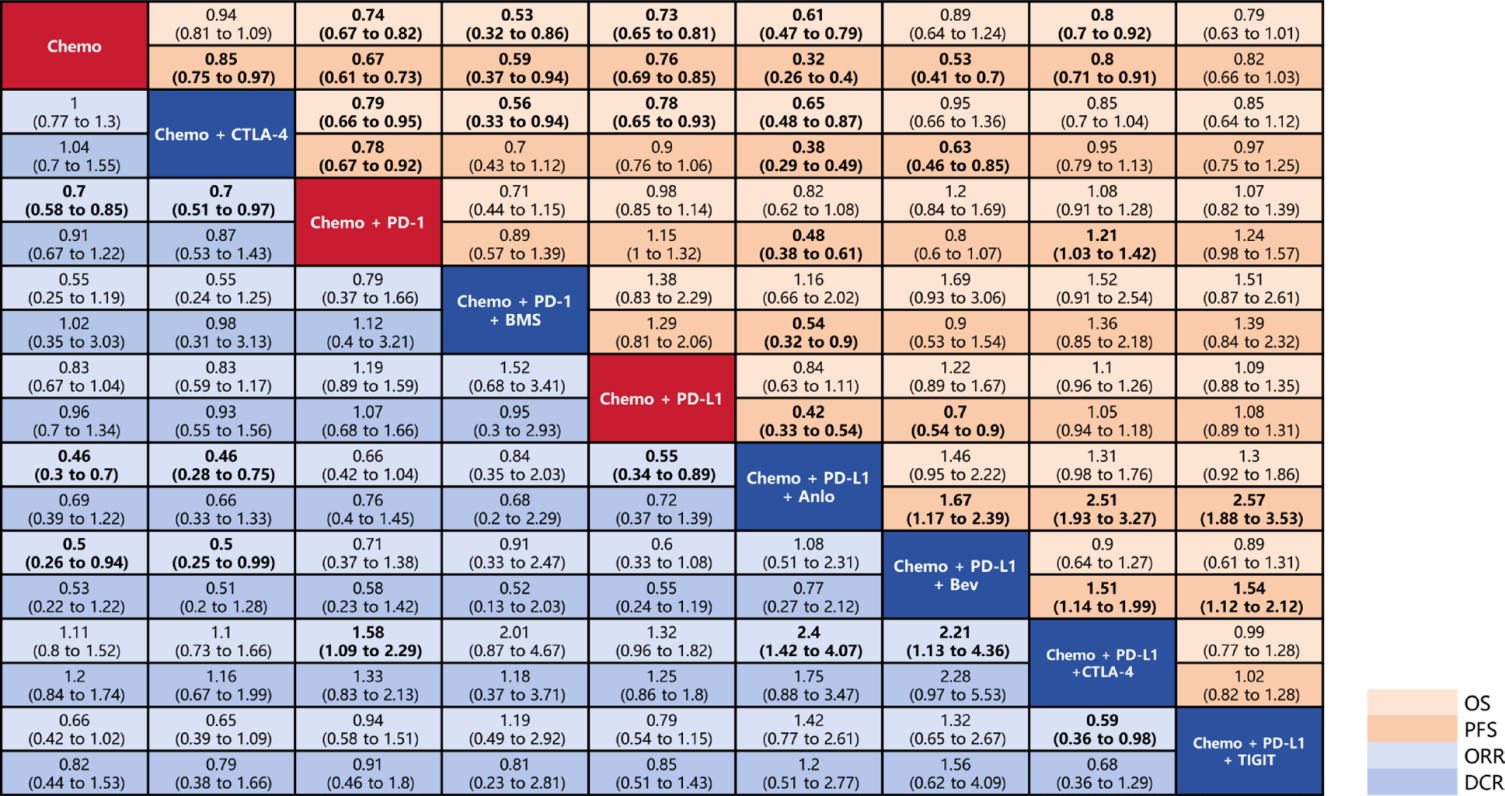
Network meta-analysis for efficacy. #1. The red squares along the diagonal represent the conventional and new first-line SOC for patients with ES-SCLC. The blue squares along the diagonal indicate experimental treatment modalities added to the SOC, which are not included in the first-line SOC. The differently colored squares on either side of the diagonal correspond to distinct clinical outcome measures, as indicated in the legend at the bottom left. All statistically significant results are shown in bold. #2. Effect sizes were considered statistically significant if the 95% confidence interval (CI) did not include 1, which was also marked. #3. A treatment modality with an HR for OS or PFS less than 1, or an OR for ORR or DCR greater than 1, was deemed more favorable. #4. Abbreviations: Chemo, Etoposide-Platinum (Cisplatin or Carboplatin) Chemotherapy; CTLA-4, Cytotoxic T-lymphocyte associated protein 4; PD-L1, programmed cell death ligand 1; PD-1, programmed cell death 1; Anlo, anlotinib; Bev, bevacizumab; SOC, Standard of Care

Figure 2 presents the clinical efficacy outcomes for all treatment modalities, including OS, PFS, ORR, and DCR. The complete league table is provided in Supplementary File 2.

Based on one of the two new first-line SOCs, the Chemo + PD-L1 regimen, the results are as follows: In terms of ORR, the Chemo + PD-L1 + Anlo regimen demonstrated significant benefits compared with the Chemo + PD-L1 regimen (OR, 1.81; 95%CI, 1.13–2.91). The Chemo + PD-L1 + Bev regimen did not result in a statistically significant improvement in ORR (OR, 1.67; 95%CI, 0.92–3.04). The complete dataset has been preserved in Supplementary File 2.

In terms of PFS, the addition of anti-angiogenic agents—whether anlotinib (HR, 0.42; 95%CI, 0.33–0.54) or bevacizumab (HR, 0.70; 95%CI, 0.54–0.90)—was beneficial for the patients. Notably, the Chemo + PD-L1 + Anlo regimen demonstrated greater PFS benefits compared with the Chemo + PD-L1 + Bev regimen (HR, 0.60; 95%CI, 0.42–0.86). Moreover, the Chemo + PD-L1 + Anlo regimen also significantly improved PFS compared with the Chemo + PD-1 regimen (HR, 0.48; 95%CI, 0.38–0.61).

In terms of OS, compared with the Chemo + PD-L1 regimen, the addition of bevacizumab, anti-CTLA-4 antibodies, or anti-TIGIT antibodies had no significant benefits, nor did they result in notable harm. (The HRs for all three comparisons > 1.) Although the Chemo + PD-L1 + Anlo regimen demonstrated an HR value ˂ 1 (HR = 0.84) for OS compared with the Chemo + PD-L1 regimen. The result was not statistically significant.

Based on a second new first-line SOC, the Chemo + PD-1 regimen, the Chemo + PD-1 + BMS regimen demonstrated HR values ˂ 1 for both OS (HR = 0.71) and PFS (HR = 0.89). However, these results did not reach statistical significance (OS, 95%CI 0.44–1.15; PFS, 95%CI 0.57–1.39).

### Network meta-analysis for the outcomes of safety

**Fig. 3:**
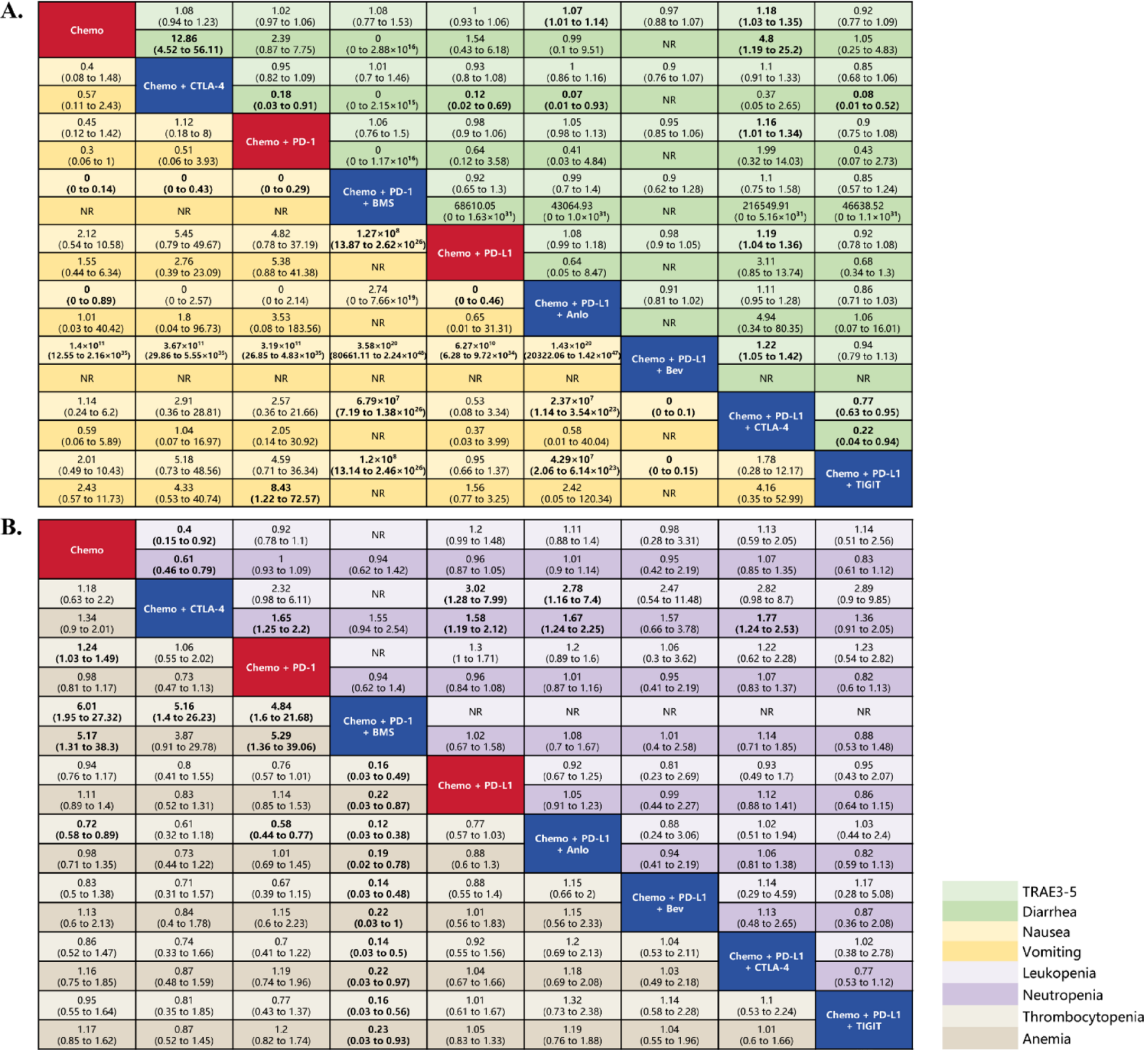
Network meta-analysis for safety. **A** Overall toxicity and gastrointestinal symptoms. **B** Hematology toxcity. #1. The red squares along the diagonal represent the conventional and new first-line SOC for patients with ES-SCLC. The blue squares along the diagonal indicate experimental treatment modalities added to the SOC, which are not included in the first-line SOC. The differently colored squares on either side of the diagonal correspond to distinct clinical outcome measures, as indicated in the legend at the bottom left. All statistically significant results are shown in bold. #2. Effect sizes were considered statistically significant if the 95% confidence interval (CI) did not include 1, which was also marked. #3. An RR greater than 1 for TRAEs suggested a higher toxicity risk. #4. When collecting data, prioritize using “Leukopenia; Neutropenia; Thrombocytopenia”. If these specific data are unavailable, use the corresponding “Decreased white-cell count; Decreased neutrophil count; Decreased platelet count” data. #5. When collecting data, if TRAEs data are not reported, use AEs (Adverse Events) data instead. #6. Abbreviations: Chemo, Etoposide-Platinum (Cisplatin or Carboplatin) Chemotherapy; CTLA-4, Cytotoxic T-lymphocyte associated protein 4; PD-L1, programmed cell death ligand 1; PD-1, programmed cell death 1; Anlo, anlotinib; Bev, bevacizumab; SOC, Standard of Care

Figure 3 illustrates the safety outcomes for all treatment modalities. The complete league table is provided in Supplementary File 2.

Compared with the Chemo + PD-L1 regimen, the Chemo + PD-L1 + CTLA-4 regimen demonstrated a significantly elevated risk of grade ≥ 3 TRAEs (RR, 1.19; 95%CI, 1.04–1.36). However, the addition of anlotinib (RR, 1.08; 95%CI, 0.99–1.18), bevacizumab (RR, 0.98; 95%CI, 0.9–1.05), or the anti-TIGIT antibody (RR, 0.92; 95%CI, 0.78–1.08), compared with the Chemo + PD-L1 regimen did not result in a statistically significant difference for risk of grade ≥ 3 TRAEs.

Interestingly, the inclusion of different anti-angiogenic agents in the Chemo + PD-L1 regimen had markedly different effects on the risk of severe nausea. The addition of bevacizumab (RR, 0; 95%CI, 0–0.16) was identified as a protective factor, while anlotinib (Chemo + PD-L1 regimen versus Chemo + PD-L1 + Anlo regimen: RR, 0; 95%CI, 0–0.46) acted as a risk factor.

In terms of hematologic toxicity, both the Chemo + PD-1 regimen (RR, 1.65; 95%CI, 1.25–2.2) and the Chemo + PD-L1 regimen (RR, 1.58; 95%CI, 1.19–2.12) exhibited a higher risk of severe neutropenia compared with the Chemo + CTLA-4 regimen. In addition, the Chemo + PD-L1 regimen also showed a higher risk of severe leukopenia compared with the Chemo + CTLA-4 regimen (RR, 3.02; 95%CI, 1.28–7.99). These findings highlight the superior advantage of the Chemo + CTLA-4 regimen in granting exemption from the toxicity of white blood cells. However, the same conclusion is not applicable when comparing the Chemo + PD-L1 + CTLA-4 regimen with the Chemo + PD-L1 regimen.

Notably, the Chemo + PD-1 + BMS regimen exhibited significantly lower risks of severe thrombocytopenia (compared with the Chemo + PD-1 regimen: RR, 0.21; 95%CI, 0.05–0.63; compared with the Chemo + PD-L1 regimen: RR, 0.16; 95%CI, 0.03–0.49) and anemia (compared with the Chemo + PD-1 regimen: RR, 0.19; 95%CI, 0.03–0.74; compared with the Chemo + PD-L1 regimen: RR, 0.22; 95%CI, 0.03–0.87) compared with the current first-line SOC.

### Rank probability and inconsistency assessment

**Fig. 4:**
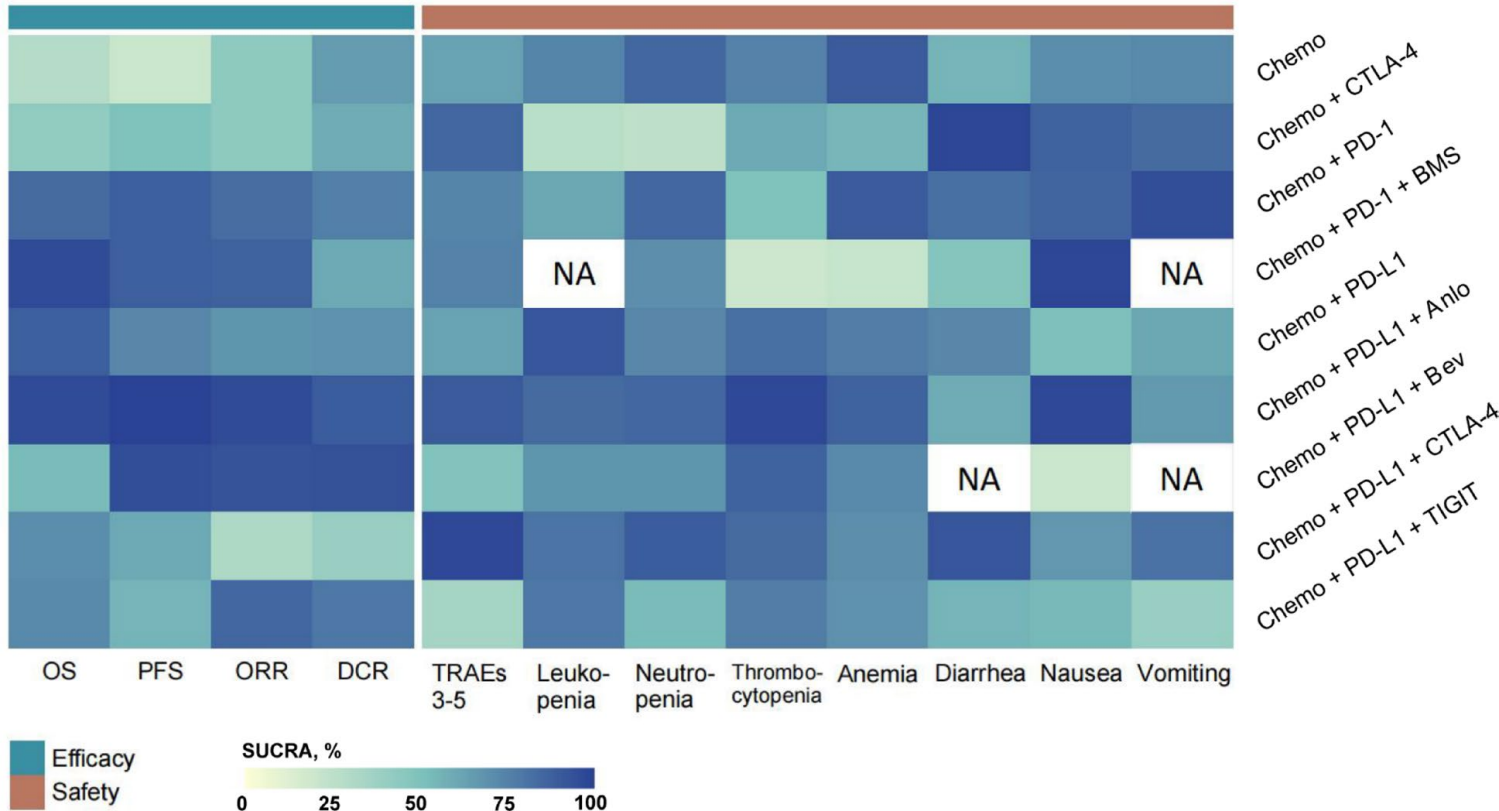
The ranking probabilities of First-Line Treatment Regimens for Patients with ES-SCLC. #1. Each cell in the heatmap is colored based on the Surface Under the Cumulative Ranking (SUCRA) value of the corresponding treatment and outcome. Blue indicates a 100% probability of being ranked first, while yellow indicates a 0% probability of being ranked first. #2. Abbreviations: NA, Not Applicable; Chemo, Etoposide-Platinum (Cisplatin or Carboplatin) Chemotherapy; CTLA-4, Cytotoxic T-lymphocyte associated protein 4; PD-L1, programmed cell death ligand 1; PD-1, programmed cell death 1; Anlo, anlotinib; Bev, bevacizumab

A heatmap (Fig. 4) was generated to visualize the comparative magnitudes of SUCRA values across treatment modalities.

Among all treatment groups, the Chemo + PD-L1 + Anlo regimen had the highest probability of ranking first for PFS (SUCRA = 0.99) and ORR (SUCRA = 0.97), while also exhibiting the highest probability of grade ≥ 3 thrombocytopenia (SUCRA = 0.97) during treatment. The Chemo + PD-L1 + Bev regimen had the highest probability of ranking first in terms of DCR (SUCRA = 0.94).

Concerning OS, the Chemo + PD-1 + BMS regimen ranked first with the highest probability (SUCRA = 0.965), followed by the Chemo + PD-L1 + Anlo regimen in second place (SUCRA = 0.964). However, the Chemo + PD-1 + BMS (SUCRA = 0.99) regimen had the highest probability of severe nausea.

Regarding the current first-line SOC for ES-SCLC, the Chemo + PD-1 regimen had the highest probability of ranking first for anemia (SUCRA = 0.91) and vomiting (SUCRA = 0.95), while the Chemo + PD-L1 regimen had the highest probability for leukopenia (SUCRA = 0.93).

Additionally, the Chemo + PD-L1 + CTLA-4 regimen was associated with the highest probability of experiencing grade ≥ 3 TRAEs (SUCRA = 0.98) and neutropenia (SUCRA = 0.90).

Additionally, Supplementary File 3 presents the ranking probabilities and SUCRA values for different treatment groups across all the clinical outcomes, including OS, PFS, ORR, DCR, leukopenia, neutropenia, thrombocytopenia, anemia, diarrhea, nausea, and vomiting. Supplementary File 4 provides the DIC, which was employed to assess the relative goodness-of-fit between fixed-effect and random-effect models.

## Discussion

Through literature retrieval and strict selection, this study ultimately included 14 head-to-head RCTs, comprising 3 Phase II trials and 11 Phase III trials. We analyzed the clinical benefit and safety data from 6,473 patients enrolled in these trials.

Consistent with conventional academic perspectives, both the Chemo + PD-L1 regimen and the Chemo + PD-1 regimen demonstrated significant improvements in OS and PFS compared with chemotherapy alone in this study. However, no significant difference in OS or PFS benefit was observed between the Chemo + PD-L1 and Chemo + PD-1 regimens.

In contrast, the Chemo + CTLA-4 regimen, another form of chemoimmunotherapy, failed to achieve superior OS over chemotherapy alone. This discrepancy may be attributed to distinct mechanisms of action. PD-1/PD-L1 inhibitors primarily function by blocking the PD-1/PD-L1 interaction, directly restoring T cell-mediated tumor killing [6, 18]. However, CTLA-4 inhibitors act predominantly by enhancing the binding affinity between CD28 on T cells and B7-1/B7-2 on antigen-presenting cells (APCs), indirectly augmenting T cell anti-tumor activity [19, 20].

The immunologically “cold” tumor microenvironment (TME) characteristic of SCLC, marked by low immune infiltration, may render CTLA-4 inhibition insufficient to activate an adequate anti-tumor immune response. PD-1/PD-L1 inhibitors, however, rely more heavily on pre-existing T cell infiltration [19–21]. Furthermore, multiple systematic reviews and meta-analyses indicate that CTLA-4 inhibitor-based combinations significantly increase the risk of grade 3–5 TRAEs compared with PD-1/PD-L1 inhibitor combinations with chemotherapy, which exhibit a more manageable toxicity profile [5, 22–24]. The findings of the present study corroborate these observations.

The results also showed that the addition of an anti-angiogenesis drug, bevacizumab or anlotinib, to the Chemo + PD-L1 regimen did not significantly improve OS but positively affected PFS. Moreover, the addition of anlotinib demonstrated superior PFS benefits compared with bevacizumab. In fact, multiple meta-analyses [5, 25, 26] have shown that, before the advent of immunotherapy, bevacizumab combined with platinum-based etoposide chemotherapy did not improve OS but did improve PFS. The introduction of immunotherapy—the Chemo + PD-L1 + Bev regimen—in comparison with the Chemo + PD-L1 regimen, produced the same results. Regarding the Chemo + PD-L1 + Anlo regimen, the original study [10] did not directly establish a treatment group for the Chemo + PD-L1 regimen, and thus direct comparison data were unavailable. However, through this network meta-analysis, an indirect comparison between the two regimens was effectively achieved. Data analysis also suggests that the Chemo + PD-L1 + Anlo regimen was associated with better ORR (81.3%), although no significant ORR improvement was observed in the Chemo + PD-L1 + Bev regimen. In toxicity studies, the Chemo + PD-L1 + Anlo regimen resulted in a higher risk of grade ≥ 3 nausea but did not significantly increase the overall reporting rate of severe TRAEs. Therefore, for SCLC patients with a high tumor burden, the Chemo + PD-L1 + Anlo regimen is an available first-line therapeutic option.

The data for the addition of another ICI to the new first-line SOC (the Chemo + PD-L1 regimen) are currently supported by two proposed regimens: the Chemo + PD-L1 + CTLA-4 and Chemo + PD-L1 + TIGIT regimens. The Chemo + PD-L1 + CTLA4 regimen is supported by the CASPIAN study [11, 27], where an anti-CTLA4 inhibitor, tremelimumab, was employed. The results showed that adding the anti-CTLA4 inhibitor to the Chemo + PD-L1 regimen did not significantly benefit either OS or PFS. However, safety data demonstrated that the inclusion of CTLA-4 inhibitors, compared with implementing the Chemo + PD-L1 regimen alone, led to increased events of severe toxicity. Tiragolumab, another ICI, is an anti-TIGIT inhibitor. Many preclinical studies [28–31] have shown that the combination of an anti-TIGIT inhibitor and a PD-L1 inhibitor, results in superior tumor-killing effects compared with using PD-L1 inhibitors alone. The molecular mechanisms underlying the anti-tumor effects of combination blockade primarily include (1) triggering Fcγ receptor (FcγR)-dependent activation of tumor myeloid cells [28]; (2) eliciting the clonal expansion of tumor antigen-specific CD8^+^ T cells driven by CD226 [29, 32]; and (3) significantly upregulating pro-inflammatory cytokines in both CD4^+^ and CD8^+^ cells, including IL-2, IFN-γ, and TNF-α [33]. However, clinical trial results—in the SKYSCRAPER-02 trial [7] or this network meta-analysis—have shown no significant benefit to OS or PFS for ES-SCLC patients treated with the Chemo + PD-L1 + TIGIT regimen. Although chemotherapy combined with dual ICIs demonstrates clear efficacy advantages over chemotherapy alone, it shows no significant benefit compared to the current SOC (the Chemo + PD-1/PD-L1 regimen). Potential explanations for this observation are as follows. First, the combination of chemotherapy with dual ICIs (e.g., PD-L1 inhibitor + CTLA-4 inhibitor) results in overlapping toxicities, significantly increasing both the incidence and severity of TRAEs. Second, The TME in ES-SCLC is typically characterized by low PD-L1 expression and profound immunosuppression [20, 21]. While PD-1/PD-L1 inhibitors can restore T-cell function by blocking the PD-1/PD-L1 axis, the addition of CTLA-4 or TIGIT inhibitors may fail to adequately overcome other dominant immunosuppressive mechanisms within the TME, such as myeloid-derived suppressor cell (MDSC) infiltration or regulatory T-cell (Treg) activity. Paradoxically, excessive immune activation could potentially exacerbate T-cell exhaustion. Third, PD-L1 expression exhibits limited predictive value for treatment response in ES-SCLC [34], and reliable biomarkers for the efficacy of CTLA-4 or TIGIT inhibitors remain elusive. Consequently, the beneficiary population for dual immunotherapy proves challenging to identify precisely, likely leading to a dilution of the overall treatment benefit in unselected cohorts. The exact reasons for the outcome and potential improvement strategies still require further investigation.

Interestingly, after calculating SUCRA for OS and ranking the results, the Chemo + PD-1 + BMS regimen ranked the highest. This was followed by the Chemo + PD-L1 + Anlo regimen, and in the third and fourth places were the Chemo + PD-L1 and Chemo + PD-1 regimens, respectively. These results demonstrate the potential of combining BMS-986012 with the first-line SOC to improve OS. Fucosylated GM1 ganglioside (Fucosyl-GM1), a glycolipid antigen, is highly expressed on the surface of SCLC cells, with a prevalence ranging from 67 to ˃ 90% [35–37]. In contrast, its expression on normal cells is relatively low. BMS-986012 is a fully human IgG1 monoclonal antibody targeting fucosyl-GM1 [9, 38], and its anti-tumor activity is mainly associated with three mechanisms: (1) antibody-dependent cell-mediated cytotoxicity, (2) antibody-dependent cellular phagocytosis, and (3) complement-dependent cytotoxicity [39]. Animal studies have shown that, compared with monotherapy, combining BMS-986012 with platinum-etoposide chemotherapy or anti-PD-1 antibody can delay tumor growth, and the enhanced effect of BMS-986012 is likely mediated through NK cells and myeloid cells [39].

The combination of radiotherapy and the first-line SoC is an important avenue for the future innovation of first-line treatment in ES-SCLC. According to a phase II single-arm study, patients who received etoposide-platinum chemotherapy combined with a kind of PD-L1 antibody, followed by sequential consolidative thoracic radiotherapy (TRT) (≥ 30 Gy in 10 fractions or ≥ 50 Gy in 25 fractions, involved-field irradiation) combined with maintenance therapy of the PD-L1 antibody, showed a median OS and PFS of 21.4 months and 10.1 months, respectively [40], which is highly encouraging. Other studies, such as the MATCH and LEAD trials, have also yielded preliminary results [41, 42]. Besides, the combination of chemoimmunotherapy with thoracic radiotherapy has shown acceptable safety [40–42]. However, the optimal timing, dosage, and fractionation scheme for radiotherapy still require further exploration.

In conclusion, the addition of anti-angiogenic drugs (anlotinib/bevacizumab), another ICI (anti-CTLA4 inhibitor, tremelimumab/anti-TIGIT antibody, tiragolumab), or the immune-related targeted new drug BMS-986012 to first-line chemoimmunotherapy regimens has not significantly improved patient survival outcomes. An exception is the addition of anti-angiogenic drugs, which have shown superiority in PFS. To date, the Chemo + PD-L1/PD-1 regimen remains the first-line SOC for ES-SCLC. The Chemo + PD-L1 + Anlo regimen has demonstrated improvements in PFS and ORR with comparable toxicity, making it a first-line option for ES-SCLC; however, it cannot fully replace the current immunochemotherapy due to a lack of OS improvement.

### Limitations

This study has several limitations. First, due to the lack of data establishing correlations with outcome measures, we were unable to perform stratified subgroup analyses based on potential prognostic factors, such as sex, age, and performance status. Second, the impact of subsequent-line therapies on survival outcomes could not be adequately assessed because of incomplete treatment records. Third, the low reporting rate of immune-related AEs (irAEs) in patients restricted the analysis to frequently reported toxicities. Fourth, some emerging therapeutic strategies—including combining radiotherapy [40] or novel immune-related targeting agents [43] (such as anti-CD27 antibodies and anti-ILT4 antibodies) with the current first-line SOC—were excluded from comparisons due to a mismatch of study types and missing key data. Finally, the date of the literature search in this study was as of November 2024, and there will inevitably be a delay in the date of the final publication in the journal due to the process of searching, graphing, writing, and proofreading. We may not be able to present the results of other relevant studies published within this time frame for this study, and we will improve this point by continuously updating at a later stage.

## Supplementary Information

Below is the link to the electronic supplementary material.

Supplementary Material 1

Supplementary Material 2

Supplementary Material 3

Supplementary Material 4

Supplementary Material 5

Supplementary Material 6

Supplementary Material 7

## Data Availability

All data generated or analyzed during this study are included in this published article.
