# Supplementary figures and images for: Multidimensional comparative evaluation of first-line therapies for extensive-stage small cell lung cancer: a systematic review and network meta-analysis of clinical efficacy and safety profiles
Source: BMC Cancer. 2025 Aug 9;25:1292. doi: 10.1186/s12885-025-14750-4 (PMC12335103; doi:10.1186/s12885-025-14750-4)

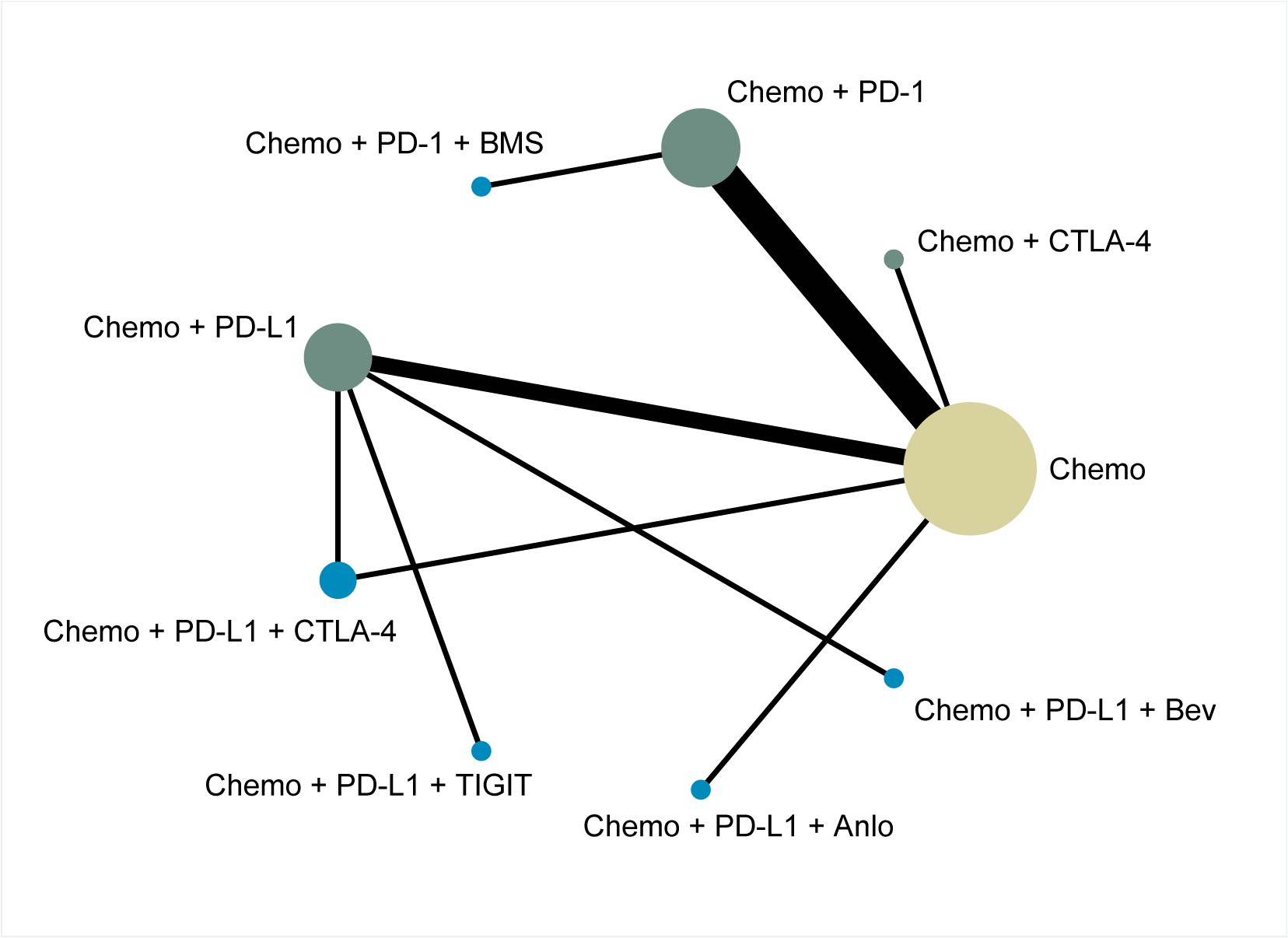

Supplement: Supplementary file 6 — Supplementary Material 6 [file 12885_2025_14750_MOESM6_ESM.tiff]

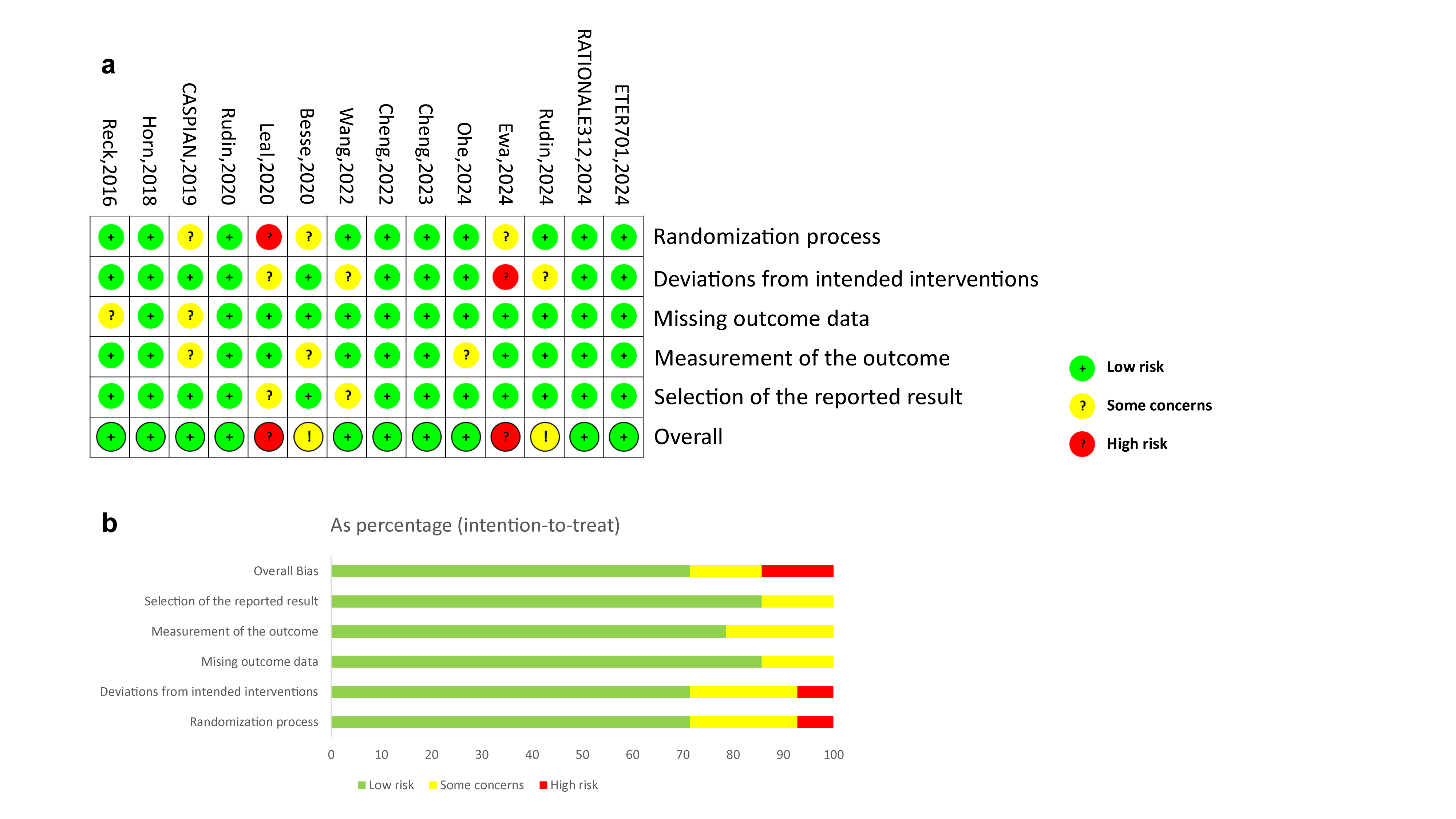

Supplement: Supplementary file 7 — Supplementary Material 7 [file 12885_2025_14750_MOESM7_ESM.tiff]
